# VITALITY trial: protocol for a randomised controlled trial to establish the role of postnatal vitamin D supplementation in infant immune health

**DOI:** 10.1136/bmjopen-2015-009377

**Published:** 2015-12-16

**Authors:** Katrina J Allen, Mary Panjari, Jennifer J Koplin, Anne-Louise Ponsonby, Peter Vuillermin, Lyle C Gurrin, Ronda Greaves, Natalie Carvalho, Kim Dalziel, Mimi L K Tang, Katherine J Lee, Melissa Wake, Nigel Curtis, Shyamali C Dharmage

**Affiliations:** 1 The Murdoch Childrens Research Institute, Parkville, Victoria, Australia; 2 The Department of Paediatrics, University of Melbourne, Parkville, Victoria, Australia; 3 The Department of Allergy and Immunology, Royal Children's Hospital, Parkville, Victoria, Australia; 4 The Centre for Epidemiology and Biostatistics, University of Melbourne, Parkville, Victoria, Australia; 5 School of Medical Sciences, RMIT University, Bundoora, Victoria, Australia; 6 Centre for Health Policy, Melbourne School of Population and Global Health, The University of Melbourne, Melbourne, Melbourne, Australia; 7 The Melbourne Children's Trials Centre (MCRI), Melbourne, Victoria, Australia

**Keywords:** EPIDEMIOLOGY, PAEDIATRICS, PREVENTIVE MEDICINE

## Abstract

**Introduction:**

Postnatal vitamin D supplementation may be associated with a reduction in IgE-mediated food allergy, lower respiratory tract infections and improved bone health. Countries in the Northern hemisphere recommend universal infant vitamin D supplementation to optimise early vitamin D levels, despite the absence of large trials proving safety or efficacy for any disease outcome. With the aim of determining the clinical and cost-effectiveness of daily vitamin D supplementation in breastfed infants from age 6–8 weeks to 12 months of age, we have started a double-blind, randomised, placebo-controlled trial of daily 400 IU vitamin D supplementation during the first year of life, VITALITY.

**Methods nd analysis:**

Infants (n=3012) who are fully breastfed and not receiving vitamin D supplementation will be recruited at the time of their first immunisation, from council-led immunisation clinics throughout metropolitan Melbourne, Australia. The primary outcome is challenge-proven food allergy at 12 months of age. Secondary outcomes are food sensitisation (positive skin prick test), number of lower respiratory infections (through hospital linkage), moderately-severe and persistent eczema (by history and examination) and vitamin D deficiency (serum vitamin D <50 nmol/L) at age 12 months. The trial is underway and the first 130 participants have been recruited.

**Ethics and dissemination:**

The VITALITY study is approved by the Royal Children's Hospital (RCH) Human Research Ethics Committee (#34168). Outcomes will be disseminated through publication and will be presented at scientific conferences.

**Trial registration numbers:**

ANZCTR12614000334606 and NCT02112734; pre-results.

**Strengths and limitations of this study**

- Allergic disease is on the rise.
- Vitamin D deficiency has been associated with the development of food allergy in infants.
- Vitamin D status appears to play an important role in the adequacy of immune responses to viral respiratory tract infections.
- Large scale trials of postnatal vitamin D for any disease outcome are lacking.
- Vitamin D supplementation could optimise infant immune health in the first year of life reducing the risk of allergic disease and respiratory diseases.

## Introduction

Vitamin D is likely to play a role in early infant immune health, with emerging evidence that early life vitamin D deficiency increases the risk of developing childhood diseases such as food allergy^1^ lower respiratory infections (LRIs)^2^ and eczema.^3^ A marked shift to vitamin D deficiency in early life in modern populations may be an underlying driver of the reported rise in allergic diseases including food allergy and eczema.

The rise in food allergy in developed countries is well documented.^1^ ^4^ ^5^ The increase is most pronounced in children under 5 years of age which suggests a causal role for early life determinants. Recent evidence suggests that low vitamin D at birth or during infancy is associated with an increased risk of food allergy^6^ and eczema.^7^ In addition food allergy and eczema prevalence is higher the further from the equator a person resides, lending support to the hypotheses that low vitamin D (through low ultraviolet (UV) exposure) may play a role in aberrant immune development in early life and increase the risk of developing food allergy and eczema.^3^ Food allergy is one of the earliest manifestations of the immune deviation,^8^ but also strongly coassociates with eczema in infancy and asthma later in life.^9^

Vitamin D supplementation in the first year of life has also been associated with a reduced risk of LRIs^10^ and reduced recovery time in infants with acute bronchiolitis.^11^ Since early life wheezy LRIs are also strongly associated with longer term respiratory outcomes including asthma^12^ understanding the role of vitamin D in the risk of early life respiratory diseases is also important.

Vitamin D deficiency (currently defined as 25OHD3<50 nmol/L) has become more common in modern communities over time, presumably due to inadequate sun exposure resulting from indoor activities and skin cancer concerns.^13^ Vitamin D deficiency is common in Victoria, Australia and also appears to be increasing among pregnant women^14^ and infants.^6^ In addition, the prevalence of infant vitamin D deficiency was fourfold higher among fully breastfed infants than fully formula fed (p<0.001) even at 12 months after solid food had been introduced.^15^

Unlike other countries in the Northern Hemisphere, Australia does not recommend universal vitamin D supplementation. Furthermore as one of the few countries that does not routinely fortify the food chain supply, Australia is uniquely placed internationally to assess the role of vitamin D in early life development.

## Objectives

With the ultimate goal of developing improved public health guidelines for vitamin D supplementation of infants, we aim to determine if vitamin D supplementation in infants from 6 to 8 weeks of age leads to a reduction in the following during the first year of life:

1. Challenge-proven food allergy
2. Lower respiratory infections
3. Food sensitisation
4. Doctor diagnosed eczema
5. Vitamin D deficiency

## Methods and analysis

### Design

VITALITY is a double-blind, randomised, placebo-controlled trial of vitamin D supplementation during the first year of life. The trial will be conducted and reported according to CONSORT^16^ guidelines with the cost-effectiveness analysis conducted and reported according to CHEERS guidelines.^17^

### Setting and participants

**Figure 1:**
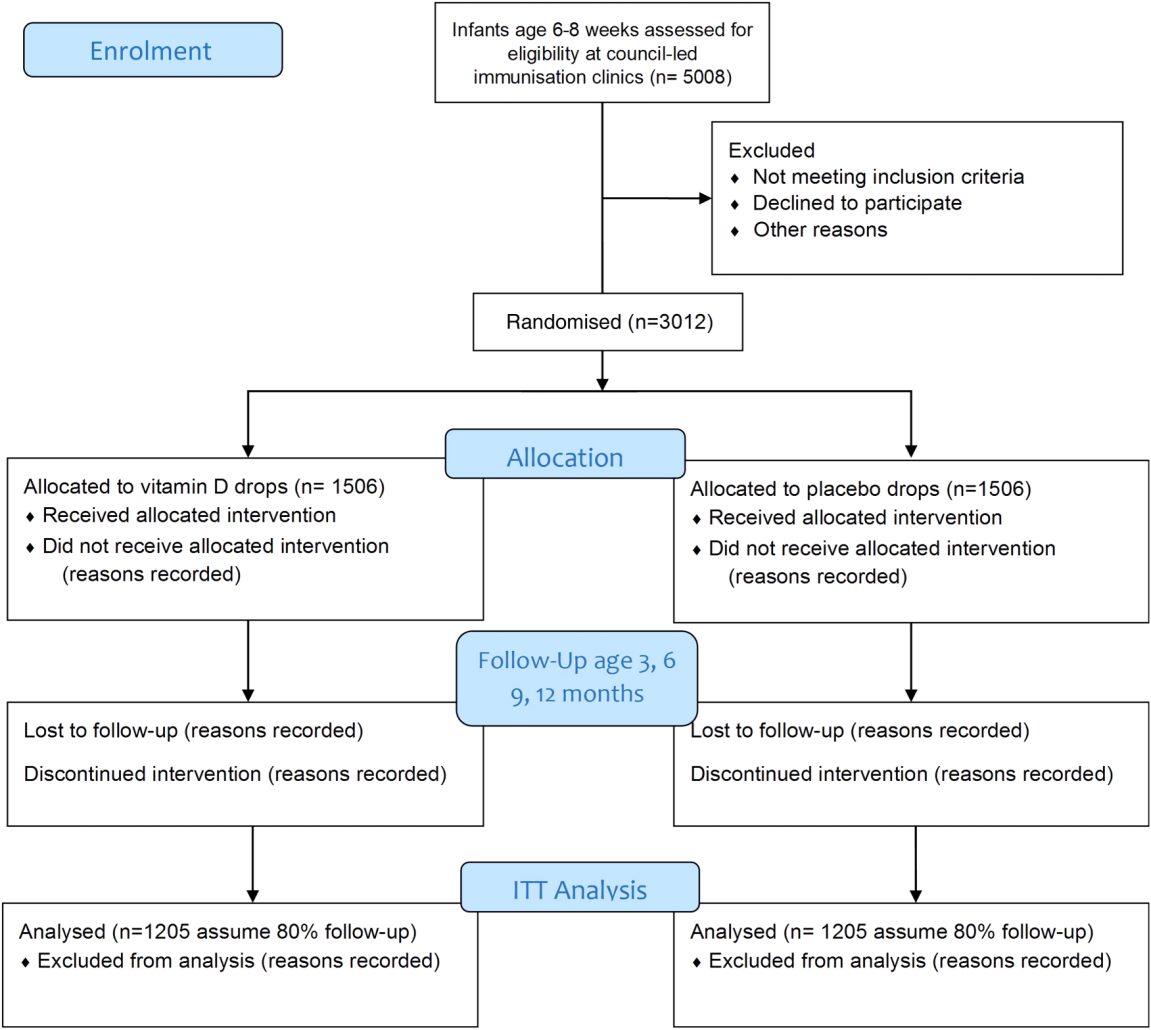
Study diagram.

The trial will be conducted in 6–8 week old breast fed infants not already receiving vitamin D supplementation. Infants will be recruited from council run immunisation sessions within Melbourne, Australia. The study diagram is shown in figure 1. We aim to recruit 1506 infants per arm, 3012 total (see sample size). The study is restricted to breast fed infants because formula feeding from birth can be equivalent to vitamin D supplementation as formula contains approximately 400 IU of vitamin D/L. The amount of formula that infants consume by age 6 months is based on body weight and amount of solid food consumed but generally can considered to be equivalent to the infant receiving <400 IU.

### Inclusion and exclusion criteria

*Inclusion criteria*: Healthy, term, breastfeeding 6–8 week old infants. Infants of mothers who intend to continue predominantly breastfeeding until 6 months will be recruited since formula feeding is a surrogate form of partial vitamin D supplementation.

*Exclusion criteria*: Infants with the following will be excluded: already receiving vitamin D supplementation, born premature (<37 weeks)/low birth weight (<2500 g), multiple births, poor health due to a current or past significant disease state or congenital abnormality or infants on medication that interfere with vitamin D metabolism. Mothers who receive antenatal vitamin D supplementation will not be excluded because in our setting, this has not been shown to be protective against postnatal infant vitamin D deficiency.^15^

### Randomisation

Randomisation will be undertaken using a web-based randomisation system set up within the trial database (REDCap). The randomisation schedule will be generated using permuted blocks of variable lengths by an independent statistician. To ensure concealment of allocation participants will only be randomised once they clearly meet inclusion criteria. Participants, their parents and all members of the study team, aside from the clinical trials pharmacist, will remain blinded to treatment allocation throughout the trial.

### Intervention

Infants will be randomly assigned to receive 400 IU vitamin D3-Cholecalciferol (1 drop, 0.03 mL) (Baby D drops) or an identical placebo (1 drop vegetable oil) daily until 12 months of age. Both active and placebo interventions will be supplied by the D Drops Company, Ontario, Canada which currently provides the active product commercially under the regulation of Health Canada's Natural Health Product Directorate.

The current daily dose recommended for infants in the first year of life is 400 IU in all available published international guidelines; USA^18^ and UK National Health Service (NHS) guidelines. A daily dose of 400 IU administered from around 4 weeks of age was sufficient to increase vitamin D levels to ≥50 nmol/L in 98% of infants by 3 months of age.^19^

### Compliance

We have developed an iPhone application to remind study participants to give their infant the study medication and to monitor dosing compliance. This information can be emailed from the app to vitality@mcri.edu.au and incorporated into the REDCapdatabase. An android-based application is in development. For participants without smart phones, a paper diary system is available. These can be sent back via mail or photographed and emailed. Participants are asked to send their empty bottles of study medication to the Clinical Trials Pharmacy for re-weighing to measure compliance with daily dosing. To date compliance with daily dosing is 90% based on the number of returned, completed diaries.

### Outcomes

*Primary*

- Challenge-proven food allergy at 12 months of age.

*Secondary*

- Number of LRIs by 12 months of age;
- Food sensitisation (positive skin prick test) at 12 months of age;
- Moderately-severe and persistent eczema at 12 months of age;
- Vitamin D deficiency at 12 months of age.

### Ethics and dissemination

The study has been approved by the Royal Children's Hospital (RCH) Human Research Ethics Committee (#34168) and is registered with the Australia & New Zealand Clinical Trials Registry (ANZCTR12614000334606) and U.S. National Institutes of Health (NCT02112734). Written informed consent will be obtained from all participants included in the trial. Participants will be informed that they are not obliged to take part in the study and are free to withdraw at any time without negative consequences on their future care. The privacy of participants will be protected and their names and personal information will be kept confidential. No serious adverse reactions are anticipated in the trial but these will be monitored by the Data Safety and Monitoring Committee. Outcomes will be disseminated through publication according to the SPIRIT statement and will be presented at scientific conferences.

### Data collection

Table 1 summarises timing of measures, described in more detail below. Web-based questionnaires will be administered to parents at baseline and when their infant is aged 3, 6, 9 and 12 months. Infants will be invited in for a clinical assessment, blood sample and allergy testing at age 12 months at the completion of the intervention. An optional RCH clinic visit at age 6 months will be offered to participants and will include an eczema examination and blood draw. Daily diaries will be used to record compliance with drug dosing, changes to infant feeding, new allergy symptoms, medication use and medical visits to inform the economic evaluation.

**Table 1:** Summary and timing of measures

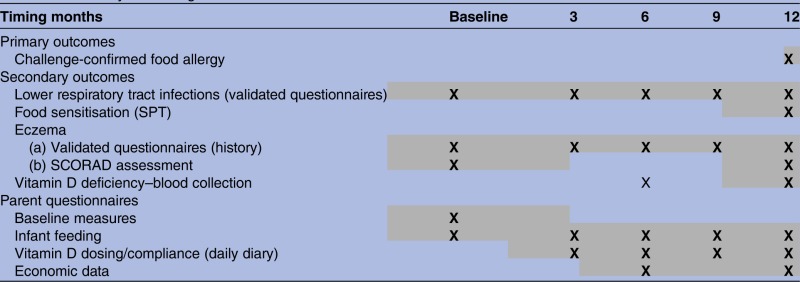

SCORAD, Scoring of Atopic Dermatitis; SPT, skin prick test.

### Primary outcome

*Challenge confirmed food allergy*: These will be undertaken in all participants with a positive skin prick test to food allergens at age 12 months. Food challenges will follow Australasian Society of Clinical Immunology and Allergy (ASCIA) guidelines as used by the RCH Allergy Clinic and validated within our HealthNuts study in which we undertook more than 1800 oral food challenges^4^ ^6^ ^20^ at age 12 months.

### Secondary outcomes

*LRIs*: The parent questionnaires will capture information on bronchiolitis, healthcare visits/hospital admissions and medication use in the past 3 months. We will access general practitioner (GP) or hospital records to obtain accurate data on lower respiratory infections.

*Food sensitisation*: Sensitisation to a panel of allergens will be assessed in all participants at 12 months of age: cow's milk, egg, peanut, cashew, sesame. Skin prick allergy testing will be performed according to standard guidelines as per previous HealthNuts protocol.^20^

*Eczema*: Eczema history will be assessed by the U.K. Working Party’s Diagnostic Criteria for Atopic Dermatitis using parent questionnaire data. Severity of eczema will be assessed using Scoring of Atopic Dermatitis (SCORAD)^21^ at baseline/12 months as follows: Mild—SCORAD <25, Moderate—SCORAD, 25–50, Severe—SCORAD ≥50. Persistent eczema will be assessed if age of onset of eczema was by 9 months and evident at age 12 months.

*Vitamin D deficiency*: Infant 25OHD3 levels and metabolites including the C3-epimer of 25OHD3 will be measured using liquid chromatography-tandem mass spectrometry.^22^ The method will be referenced to the Joint Commission for Traceability in Laboratory Medicine's recommended reference standard to provide accuracy for the vitamin D result. Participant's vitamin D status will be defined by their blood 25OHD3 level, as: severely deficient <12.5 nmol/L; moderately deficient 12.5–25 nmol/L; mild deficiency 26–49 nmol/L; sufficient ≥50 nmol/L; and elevated >220 nmol/L.^23^

*Parent Baseline Questionnaire*: To check that potential environmental and other confounding factors are balanced between the two randomisation groups, baseline parent questionnaires will collect data on: parental smoking, medication use, parental ancestry, ethnicity, education, employment, occupation, family history of allergy, use of supplements including vitamin A, D and cod liver oil, number of siblings; and pet ownership.

*Parent Questionnaire (3 monthly)*: To capture dietary vitamin D intake and environmental UV radiation (UVR) exposure, we will ask questions about maternal vitamin D supplements, infant diet, maternal and infant sun exposure and sun protective behaviours as in our previous work,^15^ for example, number of hours spent in the sun per day, during summer or winter, weekends and holidays, will be collected for mother and child. Cumulative UVR dose will be calculated as the product of time spent outdoors and ambient UVR during the period. Composite skin exposure and sun protection indices will be measured. We have established that composite maternal UVR dose (OR 0.50 (95% CI 0.37 to 0.69) p<0.001) and maternal skin exposure index (OR 0.81 (95% CI 0.66 to 0.98) p=0.03) were associated with breastfed infant vitamin D status but infant time in the sun during the first year was not.^15^ Infant feeding (amount of breastfeeding, formula feeding and type of formula, introduction of solid foods) will also be recorded. At 12 months only, infant skin/hair/eye colour will be assessed using a standardised, validated chart. We have validated that the charts predict (p<0.001) skin melanin density by spectrophotometer.^24^

*Blood samples (12 months)*: Peripheral blood mononuclear cells will be separated from whole blood and stored in liquid nitrogen, and plasma will be stored and frozen in our −80°C freezer. These will be retained for future analysis to evaluate immune function including regulatory T cell and cytokine assays.

*Newborn screening (Guthrie) card*: consent will be obtained at recruitment to access Guthrie cards to undertake assessment of vitamin D levels at birth.

### Economic evaluation

The economic evaluation will take on a societal perspective, including both costs to the health system and patient out-of-pocket costs. It will model the potential cost-effectiveness of routine infant vitamin D supplementation compared to no supplementation, assuming Australia-wide implementation. The evaluation will assess the differential impact of supplementation on primary trial outcomes at 12 months compared with no supplementation. Costs associated with the intervention and use of healthcare services including hospital presentations and admissions, outpatient visits, visits to specialists and other providers (including alternative providers) and medications will be collected at 6 and 12 months to specifically capture costs associated with allergic symptoms that may arise in both groups. We will supplement this data by linking to the Australian Pharmaceutical Benefits Scheme (PBS) and the Medical Benefits Scheme (MBS) to obtain costs of pharmaceuticals and Medicare health service utilisation. This data will enable us to collect information about specialist visits, GP visits and medications-related costs for children with the primary and secondary outcomes identified in the study, including LRIs. This will be supplemented by costing data from the Royal Children's Hospital for inpatient and outpatient stays, in addition to the parent questionnaire from which we will gather out-of-pocket costs for time off work, caregiver time and travel time to appointments.

Results will be presented in terms of cost per case of food allergy averted. A sensitivity analysis will assess the robustness of results to the uncertainty of model parameters. We will model the longer term costs and effects of primary trial outcomes beyond the horizon of the trial; drawing from published literature, with the potential to extend the analysis to secondary outcomes determined to be clinically important as the trial progresses and related diseases, including bone health.

### Statistical analysis

Outcomes will be compared between the vitamin D and the placebo groups using an intention-to-treat analysis. For all outcome measures, the magnitude of between-group differences will be quantified by calculating estimated OR with 95% CI for the corresponding population OR. As a secondary analysis, the analysis will be repeated in the ‘per protocol’ (infants breastfed for at least 6 months and fully compliant with the randomisation and on the basis of 25OHD3 levels achieved). Further analyses using multivariable logistic regression will be undertaken to explore the sensitivity of the estimated ORs to covariate imbalance between the groups due to potential confounding factors introduced postrandomisation, such as age of onset of formula feeding. While the design mitigates very strongly against covariate imbalance between the trial arms, colleagues have recently shown that post hoc adjustment of the intervention–outcome association for such imbalances can, under some circumstances, maintain unbiased estimates while reducing their SE.^25^ Linear regression will be used for non-categorical variables.

### Sample size

A final sample size of 1205 infants in each group (2410 total), will enable us to identify a 30% reduction in food allergy with 80% power (based on a two-sided test with α=0.05). A 30% reduction in the prevalence of food allergy attributed to vitamin D supplementation represents a clinically important reduction on a population level. Assuming 80% follow-up at 12 months of age, we plan to recruit a total of 3012 participants over 3.5 years (approximately 79 per month).

### Start of the study

The trial started in December 2014, with n=130 participants as of June 2015. Of those eligible 48%% consented to participate. To date 13% have dropped out, on average within the first 6 weeks (5.9±4.0, weeks, mean±SD, min 0.4-max 15.4 weeks) after recruitment (main reasons include: difficult to give daily drops, too busy to comply with the study procedures). There have been no reported adverse events. Approximately half approached have an iPhone and 63% of those have activated use of the VITALITY iPhone drug compliance app. Table 2 shows demographic information for the first 130 participants to be enrolled.

**Table 2:** Baseline demographics for first participants n=130*

Characteristic	
Maternal age mean±SD (minimum–maximum)	33.7±4.6 year (19.4–45.5)
Paternal age mean±SD (minimum–maximum)	36.3±5.5 years (21.0–50.0)
Baby age mean±SD (minimum–maximum)	7.4 weeks±1.2 (6.0–11.5)
Maternal education n (%)	
Year 12	10 (9.0)
Trade certificate	9 (8.3)
University degree	56 (51.8)
Higher degree (eg, PhD, masters)	28 (25.9)
Other	5 (4.6)
Paternal education	
Year 12	14 (13.0)
Trade certificate	25 (23.1)
University degree	45 (41.7)
Higher degree (eg, PhD, masters)	22 (20.4)
Other	2 (1.8)
Marital status	
Single	1 (1.0)
Married	91 (84.3)
Defacto	14 (12.07)
Separated	1 (1.0)
Other	1 (1.0)
Maternal country of birth	
Australia	79 (73.1)
Other country	29 (26.8)
Paternal country of birth	
Australia	70 (65.0)
Other country	38 (35.0)
Family history of asthma	
No	54 (50.0)
Yes	54 (50.0)
Family history of eczema	
No	61 (57.0)
Yes	44 (41.1)
Do not know	2 (1.9)
Family history of food allergy	
No	75 (70.0)
Yes	30 (28.1)
Do not know	2 (1.9)
Maternal smoking	
No	104 (97.2)
Yes	3 (2.8)
Sex of baby	
Female	48 (45.7)
Male	57 (54.3)
Baby country of birth	
Australia	105 (100.0)
Feeding some formula	
No	64 (64.0)
Yes	36 (36.0)
If feeding some formula, infant age when started, weeks	
Mean±SD (minimum–maximum)	2.5±3.1 (0.0–12.0)
If feeding some formula, times given since birth, days	
Mean±SD (minimum–maximum)	16.0±18.3 (1.0–71.0)

Withdrawn from study n=19.

*Complete data set for n=108 mothers and n=105 infants.

## Data and safety monitoring

An independent Data Safety and Monitoring Committee (DSMC) consisting of at least one Statistical Society of Australia Inc (SSAI)-Accredited statistician, one consultant paediatric allergist and one external trials expert will monitor participant safety including unexpected adverse events which will be reported to the committee as they occur. Serious adverse events will be reported to the Royal Children's Hospital Human Research Ethics Committee. We will monitor the current literature including changes in Australian guidelines for infant vitamin D dosing and send a quarterly report to the DSMC who will make decisions regarding any requirements for trial discontinuation. We will not be monitoring serum 25(OH)D for safety reasons nor considering the association between 25(OH)D and outcome frequency given that 400 IU has been shown to be safe in term babies,^19^ ^26^ preterm infants^27^ and is the current recommended dose in the USA, Canada, the UK and Europe.^18^

## Discussion

The results of the VITALITY trial will determine whether vitamin D supplementation has a role in optimising infant immune health in the first year of life, reducing the risk of allergic disease and respiratory diseases. Furthermore findings will provide evidence to inform guidelines for vitamin D supplementation of infants. This will be the first population-based trial of postnatal infant vitamin D supplementation sufficiently powered to address the impact on any disease outcome including allergic disease, LRIs and bone health.

The strengths of VITALITY are that it is a large preventative, population-based trial thus the results are more likely to be generalisable. Another strength is that VITALITY will examine multiple important outcomes including food allergy and lower respiratory infections. Furthermore we are uniquely placed internationally to assess the role of vitamin D in early life development since Australia does not currently recommend universal infant vitamin D supplementation and is one of the few countries where there is no routine fortification of the food chain supply.

A logistic issue for VITALITY is that the trial needs to be large and thus costly, as the outcomes of interest are low in prevalence. Restricting the trial to infants with vitamin D deficiency would require a smaller trial, however it would be unethical to randomise these infants to placebo thereby not treating with vitamin D. The use of vitamin D supplements in the placebo group is another potential issue however we are collecting sufficient data regarding feeding practices, supplement use and sun exposure to account for this.

There are several plausible biological mechanisms for the role of vitamin D in early infant, immune health. The vitamin D receptor is widely expressed in the immune system including T cells. In particular, it promotes the expression of Interleukin-10 (IL-10) secreting T regulatory cells (crucial for maintaining immune tolerance) and potentially plays a key role in the induction of tolerance in food-allergic individuals. Vitamin D metabolites also contribute to innate epithelial defences by stimulating production of antimicrobial proteins (eg, cathelicidins and defensins).^14^ Vitamin D status appears to play an important role in the adequacy of immune responses to viral lower respiratory infections. It acts by inducing antimicrobial peptides in epithelial cells, neutrophils and macrophages^28^ and increases transcription of the innate immune protein (hCAP-18) which is capable of killing a wide variety of viral and bacterial pathogens.^29^ These findings are supported by the observation that vitamin D receptor polymorphisms (which decrease the bioavailability of vitamin D to the cell) are associated with more severe bronchiolitis^30^ and that defective control of vitamin D receptor-mediated cell signalling predisposes to severe respiratory syncytial virus bronchiolitis.^31^

## Conclusions

International enthusiasm for supplemental vitamin D has outpaced available evidence on its effectiveness^32^ and in fact argument for routine supplementation is in equipoise. If found to be both efficacious and cost-effective, vitamin D infant supplementation would provide a readily available, low harm intervention that would optimise infant immune health in the first year of life and significantly reduce the population burden of allergic disease. The VITALITY trial will also enable a future evaluation of whether postnatal vitamin D supplementation is associated with improved bone health in later childhood—the historical basis of widespread postnatal vitamin D supplementation, which remains essentially untested.
